# Association of single-nucleotide polymorphism of melanocortin-4 receptor with carcass traits in commercial rabbits under tropical conditions

**DOI:** 10.14202/vetworld.2025.2626-2635

**Published:** 2025-09-06

**Authors:** A. Setiaji, F. T. Kamila, F. Mustofa, D. A. Lestari, M. H. Kamalludin, S. Sutopo

**Affiliations:** 1 Department of Animal Science, Faculty of Animal and Agricultural Science, Universitas Diponegoro, Jl. Prof Jacub Rais, Tembalang Campus, Semarang, 50275, Central Java, Indonesia; 2 Institute of Tropical Agriculture and Food Security, Universiti Putra Malaysia, Serdang, Selangor, 43400, Malaysia

**Keywords:** carcass yield, marker-assisted selection, melanocortin-4 receptor, rabbit breeding, single-nucleotide polymorphism, tropical livestock

## Abstract

**Background and Aim:**

Rabbits are increasingly valued in tropical livestock systems for their efficient feed conversion, high-quality meat, and adaptability to small-to-medium scale farming. Genetic selection using molecular markers, such as single-nucleotide polymorphisms (SNPs) in candidate genes, offers a powerful tool to enhance carcass yield and meat quality. The melanocortin-4 receptor (*MC4R*) gene, a regulator of energy balance and feed intake, has been associated with growth and carcass traits in various livestock but remains underexplored in tropical rabbit production systems. This study aimed to investigate the association between MC4R SNPs and economically important carcass traits in three commercial rabbit breeds, New Zealand White (NZW), Hyla, and Hycole, raised under tropical conditions in Indonesia.

**Materials and Methods:**

Thirty-five male rabbits (10 NZW, 11 Hyla, 14 Hycole; aged 3–4 months) were selected from 621 bucks. DNA was extracted from blood samples, and a 127-base pair MC4R fragment was amplified by polymerase chain reaction and sequenced for SNP detection. Carcass traits measured included hot carcass weight, cold carcass weight, reference weight, carcass percentage, meat-to-bone ratio, and five commercial cut points (CP1–CP5). Association analysis between MC4R genotypes (AA, AG, GG) and carcass traits was performed using a general linear model, considering breed and genotype × breed interactions.

**Results:**

An SNP at position 519 (G>A) produced three genotypes. GG-genotype rabbits exhibited the highest carcass percentage (57.0%) and superior CP2 weight (0.12 ± 0.03 kg) and percentage (10.67 ± 1.53%), with significant genotype effects (p < 0.05) for CP2 weight and CP1 percentage. Breed effects were significant for carcass percentage and CP1 percentage (p < 0.05), while genotype × breed interactions significantly influenced CP2 traits (p < 0.01). The G allele showed a positive association with forequarter meat yield, suggesting its utility in marker-assisted selection (MAS).

**Conclusions:**

MC4R polymorphism at position 519 is significantly associated with key carcass traits in commercial rabbits under tropical conditions. The GG genotype is linked to improved carcass yield, particularly in CP2 traits, although effects vary by breed. Incorporating this SNP into MAS programs can enhance meat production efficiency and carcass quality in tropical rabbit breeding.

## INTRODUCTION

Rabbits are increasingly recognized as a valuable livestock species because of their efficiency in converting feed into high-quality animal protein. Rabbit meat is particularly notable for its favorable nutritional profile, making it an attractive alternative to traditional red meats, such as beef, lamb, and pork [1]. On average, rabbit meat contains 20%–21% high biological value protein, is low in fat, and is rich in essential nutrients. It also contains significant proportions of unsaturated fatty acids, especially oleic and linoleic acids, which contribute to cardiovascular health. Furthermore, rabbit meat provides essential minerals, such as potassium, phosphorus, and magnesium, while maintaining low levels of cholesterol and sodium, which are characteristics that support its inclusion in diets focused on heart health and weight management [2, 3]. In addition to its nutritional properties, rabbit meat demonstrates good oxidative stability and functional characteristics, which contribute to its shelf life and sensory qualities, making it suitable for both fresh consumption and processed meat products [4, 5]. Given its nutritional potential, enhancing the genetic traits that influence meat quality has become an important focus in rabbit breeding programs.

In many developing countries, including Indonesia, rabbits are raised under various production systems ranging from backyard farming to semi-intensive and intensive commercial enterprises. These systems are often small-to-medium scale and serve two purposes: providing a sustainable source of animal protein for local consumption and generating additional household income. Rabbits are ideal for such systems because of their adaptability, short reproductive cycle, and low maintenance costs [6, 7]. Several exotic breeds have been introduced in Indonesia to improve the productivity of local rabbit farming. The New Zealand White (NZW), Hyla, and Hycole are the most commonly used breeds for commercial meat production. These breeds are known for their rapid growth rates, high reproductive efficiency, and favorable carcass traits, such as high dressing percentage and lean meat yield [8, 9]. Their performance under diverse environmental conditions has made them popular choices for expanding commercial rabbit farming initiatives in tropical and subtropical regions. As rabbit farming continues to develop, there is a growing need to apply genetic improvement strategies that support enhanced growth, feed efficiency, and meat quality.

The application of molecular genetics in livestock breeding has opened new opportunities to enhance economically important traits through marker-assisted selection (MAS). Melanocortin-4 receptor (*MC4R*) is a gene of particular interest in growth and meat production. MC4R is a member of the G-protein-coupled receptor family and is primarily expressed in the hypothalamus, where it plays a critical role in regulating energy balance, appetite, and body weight. The gene influences multiple metabolic processes, including feed intake, glucose homeostasis, insulin sensitivity, fat deposition, and thermogenesis [10, 11]. MC4R has become a candidate gene for genetic selection in several livestock species due to its broad impact on growth and energy metabolism. The identification and utilization of single-nucleotide polymorphisms (SNPs) within the *MC4R* gene offer the potential to enhance selection precision, thereby accelerating genetic progress for traits such as growth rate, carcass yield, and meat composition. However, the success of such genetic approaches depends on several factors, including the density of genetic markers, the statistical models used for association analysis, the heritability of target traits, and potential interactions between genes and environmental conditions [12]. Traits with high heritability can be effectively selected using fewer markers and simpler models, whereas traits with low heritability may require more sophisticated tools and data for accurate selection.

Although previous studies by Zhang *et al*. [13] and El-Sabrout and Aggag [14] rabbits and other livestock have demonstrated associations between MC4R polymorphisms and traits such as body weight, growth rate, and fat deposition, most of this research has been conducted in temperate regions and focused on European or specialized genetic lines. Limited attention has been given to commercial rabbit breeds reared under tropical conditions, where environmental stressors, feed resources, and management systems differ significantly from those in temperate climates. Such differences can influence gene expression, genotype–phenotype relationships, and overall carcass performance. Moreover, while the role of MC4R in growth and feed intake regulation is well established, its specific association with detailed carcass characteristics, including hot carcass weight, cold carcass weight, reference weight (RW), meat-to-bone ratio, and individual commercial cut points (CP1–CP5), remains poorly documented in tropical production systems. In particular, there is a lack of breed-specific analysis examining whether MC4R effects are consistent across different commercial breeds such as NZW, Hyla, and Hycole, which are widely used in Indonesia’s rabbit industry. Addressing these gaps is essential for developing effective MAS strategies that are tailored to the unique production environments and market demands of tropical rabbit farming.

The present study aimed to investigate the association between a SNP in the *MC4*R gene (position 519 G>A) and economically important carcass traits in three commercial rabbit breeds–NZW, Hyla, and Hycole–raised under tropical conditions in Indonesia. Specifically, we sought to: (i) Identify the MC4R genotypes present in these breeds; (ii) assess their relationship with carcass parameters, including carcass percentage and the weights and percentages of five anatomically defined commercial CPs; and (iii) determine whether genotype–phenotype associations vary between breeds, indicating possible genotype × breed interactions. By clarifying the genetic influence of MC4R on carcass yield and composition in tropical rabbits, this research provides a foundation for incorporating MC4R-based selection into breeding programs, ultimately aiming to enhance meat production efficiency and quality in sustainable tropical rabbit farming systems.

## MATERIALS AND METHODS

### Ethical approval

All animal procedures were conducted in accordance with institutional and national guidelines for the care and use of animals in research and were approved by the Animal Research Ethics Committee of the Faculty of Animal and Agricultural Sciences, Universitas Diponegoro (No. 59–01/A-01/KEP-FPP).

### Study period and location

The study was conducted from February to May 2024 at the teaching and research farm of Universitas Diponegoro.

### Animal selection and management

A total of 35 male rabbits (aged 3–4 months, weight 2.10 ± 0.17 kg were randomly selected from 621 bucks at Temanggung Rabbit Farm, Temanggung Regency, Central Java, Indonesia. The sample consisted of 10 NZW, 11 Hyla, and 14 Hycole. The selected rabbits were housed in a closed house, separated by individual cages (30 × 50 × 40 cm³). Although the housing was enclosed, natural ventilation was supported by exhaust fans to maintain air circulation under humid conditions. They were given access to drinking water *ad libitum* and fed commercial pelleted feed twice daily (08:00 and 16:00 h) (16% crude protein, 12% moisture, 2% crude fat, 14% crude fiber, and 0.5% calcium). The rabbits were slaughtered with an average body weight of 2.10 ± 0.17 kg.

### Carcass processing and definition of traits

The rabbits were fasted for 12 h and weighed before slaughter. The protocols of the Rabbits Science Association were followed for slaughtering and carcass traits [15]. The hot carcass weight, cold carcass weight, RW, and CP1–CP5 weight were among the parameters that were measured. After slaughter, each carcass was bled and skinned, and the genitals and urinary bladder organs were removed. The carcasses, including the head, thoracic cage organs (heart, lungs, thymus, trachea, and esophagus), liver, kidneys, and perirenal and scapular fat, were weighed and referred to as hot carcass weight. The carcasses were chilled at 4°C for 8 h, and the weight of the cold carcass was recorded. The head, liver, lungs, thymus, esophagus, heart, and kidneys were removed to obtain the RW, which only contained meat, fat, and bone. All carcass processing and dissections were performed by the same technician to ensure consistency.

The commercial CPs were defined as follows: The section between the 7^th^ and 8^th^ thoracic vertebra following the prolongation of the ribs when cutting the thoracic wall (CP1); the section between the last thoracic and the first lumbar vertebra, following the prolongation of the 12^th^ rib when cutting the thoracic wall (CP2); the section between the 6^th^ and 7^th^ lumbar vertebra, cutting the abdominal wall transversally to the vertebral column (CP3); separation of forelegs including insertion and thoracic muscles (CP4); and separation of hind legs, including *os coxae* and posterior part of pars lateralis and pars medialis (CP5).

According to Blasco and Ouhayoun [15], the calculation formula for carcass/dressing out percentage, commercial cut percentage, and meat-to-bone ratio was as follows:

Carcass percentage = (Cold carcass weight/Slaughter weight) × 100%

Where cold carcass weight refers to the carcass weight after chilling at 4°C for 8 h, and slaughter weight is the live body weight of the rabbit after 12 h of fasting.

Commercial cut percentage = (Commercial cut weight/Cold carcass weight) × 100%

The commercial cuts (CP1–CP5) were defined based on anatomical separation points, as detailed in the methods section.

Meat–bone ratio = (Left hind leg meat weight/Left hind leg bone weight)

This ratio reflects muscle development relative to the hind leg’s bone structure.

### DNA isolation

Before slaughtering, 3 mL of blood was obtained from the marginal vein (ear vein), collected into an ethylenediaminetetraacetic acid tube, and kept chilled in ice. All samples were immediately transported to the laboratory on ice and stored at −20°C until DNA extraction. The DNA was extracted using a Gene JET Whole Blood Genomic DNA Extraction Kit (Thermo Fisher Scientific, Waltham, MA, USA) according to the manufacturer’s protocol. DNA quality and integrity were confirmed using 1% agarose electrophoresis with ethidium bromide staining.

### Polymerase chain reaction (PCR) amplification and SNP identification

PCR was performed in a 50 μL volume using myTaq red mix (Thermo Scientific, San Francisco, CA, USA). The primers provided by Fontanesi *et al*. [16] are listed in Table 1, amplifying the 127-base pair (bp) product size (5′-flanking region and exon 1). The total PCR reaction volume was 50 μL, containing 25 μL Taq Polymerase, 19 μL ddH_2_O, 1 μL forward primer, 1 μL reverse primer, and 4 μL of template DNA.

**Table 1:** List of PCR primers used in this study [16].

No.	Primer type	Sequence	Product size
1	Forward	5’- CATGAACTCCACCCACCAC-3’	127 bp
2	Reverse	5’- CTCATAGCACCCTCCATCAGACTAG-3’	

PCR = Polymerase chain reaction, bp = Base pair

The PCR program was performed using a T100 Bio-Rad thermal cycler (Bio-Rad, CA, USA) in four main steps: (1) Pre-denaturation at 95°C for 1 min, (2) denaturation at 95°C for 15 s, (3) annealing at 56.4°C for 15 s, and (4) extension at 72°C for 55 s for 35 cycles. The amplified DNA samples were subjected to electrophoresis using 1% agarose gel for 20 min at 60 V. The quality of the PCR products was visualized using the Gel Documentary System (Labnet, San Francisco, USA). The PCR products were sequenced by Genetika Science (Tangerang, Indonesia) to determine the SNP position. MEGA version 11 (https://www.megasoftware.net/) was used for genotyping [17]. The DNA analysis stage was performed by aligning the DNA sequence by comparing the sample with the NCBI GenBank database. Alignment was performed using ClustalW (https://www.genome.jp/tools-bin/clustalw) multiple alignment in MEGA version 11 software. The sequencing results obtained were followed by a blasting process using the online Basic Local Alignment Search Tool. The total length of the MC4R product is 1,689 bp (Gene ID: 127492371).

### Statistical analysis

The association between the genotypes of the *MC4R* gene and carcass traits was analyzed using the general linear model (GLM) procedure of the Statistical Analysis System (SAS) OnDemand for Academics, SAS Institute, 2021 [18]. Before the analysis, the assumptions of normality and homoscedasticity were tested and met. Tukey’s additional test was performed when the association analysis was statistically significant (p < 0.05). The GLM statistical model was:

Y_ijk_ = μ + B_i_ + G_i_ + (G × B)_ij_ + ε_ijk_

Where Y_ijk_ is the dependent variable, μ is the overall mean of the observations, B_i_ is the effect of the i-th rabbit breed (i = 1–3), Gj is the effect of the j-th genotype (j = AA, AG, GG), (G × B)_ij_ is the interaction effect between genotype and breed, and ε_ijk_ is the random error.

## RESULTS

### Identification of MC4R polymorphism

**Figure 1:**
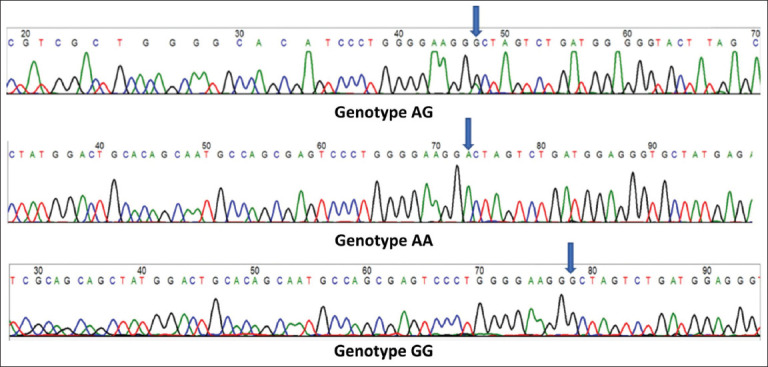
Melanocortin-4 receptor gene genotypes in commercial rabbit breeds.

Based on the sequence analysis of the *MC4R* gene, a SNP was identified at position 519, resulting in a guanine-to-adenine substitution (519G→A). This SNP produced three distinct genotypes: AA, AG, and GG (Figure 1). The sequence chromatograms clearly displayed homozygous (AA and GG) and heterozygous (AG) genotypes.

### Association of MC4R genotypes with carcass traits

The association between MC4R genotypes and carcass traits was analyzed in three commercial rabbit breeds: NZW, Hyla, and Hycole. Table 2 presents the genotype distribution across the breeds and their relationship with carcass characteristics. The genotype significantly influenced several carcass traits, particularly hot carcass weight, retail weight, and CP.

**Table 2:** Genotype-wise carcass trait means (mean ± SD).

Carcass traits	Genotype		

	AA (n = 8)	AG (n = 21)	GG (n = 6)
Hot carcass	1.19 ± 0.11	1.26 ± 0.11	1.26 ± 0.10
Cold carcass	1.14 ± 0.09	1.21 ± 0.11	1.22 ± 0.10
RW	0.92 ± 0.08	0.99 ± 0.09	0.97 ± 0.09
CP1 weight	0.28 ± 0.03	0.28 ± 0.03	0.26 ± 0.03
CP2 weight	0.09 ± 0.01^a^	0.11 ± 0.02^ab^	0.12 ± 0.03^b^
CP3 weight	0.18 ± 0.03	0.19 ± 0.02	0.19 ± 0.03
CP4 weight	0.17 ± 0.03	0.16 ± 0.02	0.16 ± 0.01
CP5 weight	0.38 ± 0.03	0.41 ± 0.05	0.41 ± 0.04
Carcass percentage	56.44 ± 2.76	56.75 ± 3.84	57.00 ± 3.35
CP1 percentage	26.18 ± 1.71^a^	24.28 ± 2.05^ab^	23.21 ± 2.23^b^
CP2 percentage	8.89 ± 0.82^a^	9.85 ± 1.04^ab^	10.67 ± 1.53^b^
CP3 percentage	17.12 ± 1.77	17.16 ± 1.22	17.01 ± 2.02
CP4 percentage	16.06 ± 2.47	14.82 ± 1.70	14.89 ± 1.54
CP5 percentage	37.27 ± 1.52	37.12 ± 4.22	38.02 ± 2.24
Meat bone ratio	7.24 ± 1.06	8.08 ± 1.24	7.92 ± 1.41

CP1 = Cut point 1, CP2 = Cut point 2, CP3 = Cut point 3, CP4 = Cut point 4, CP5 = Cut point 5, different superscript letters in the same row indicate significance (p < 0.05). SD = Standard deviation, RW = Reference weight

Rabbits carrying the AG and GG genotypes exhibited a higher average hot carcass weight than those with the AA genotype. The AG genotype had the highest retail weight, suggesting better muscle development or meat yield. Individuals with the GG genotype demonstrated the highest carcass percentage, averaging 57%, which was notably higher than those with AA and AG genotypes. These findings suggest that the G allele contributes positively to meat deposition and carcass yield in rabbits.

### Genotype effects on commercial CPs

Table 3 presents further analysis of the association between MC4R genotypes and CP traits. Significant genotype effects (p < 0.05) were observed for CP2 weight and CP1 percentage, indicating that genetic variation at the MC4R locus influences these traits. In addition, the genotype significantly influenced the percentage of CP2 (p < 0.01), reinforcing the role of this polymorphism in determining specific carcass parts.

**Table 3:** Association of *MC4R* with carcass traits in three rabbit breeds (NZW, 11; Hyla, 10; Hycole, 14).

Carcass traits	Effect	f value	p-value
Hot carcass	Breed	0.44	0.646
	Genotype	0.37	0.696
	Genotype (breed)	1.92	0.137
Cold carcass	Breed	1.15	0.331
	Genotype	0.39	0.682
	Genotype (breed)	2.03	0.119
RW	Breed	1.57	0.227
	Genotype	0.57	0.574
	Genotype (breed)	2.40	0.076
CP1 weight	Breed	1.57	0.227
	Genotype	0.57	0.574
	Genotype (breed)	2.40	0.076
CP2 weight	Breed	2.34	0.116
	Genotype	3.97*	0.031
	Genotype (breed)	4.66**	0.006
CP3 weight	Breed	2.09	0.143
	Genotype	0.40	0.672
	Genotype (breed)	0.76	0.564
CP4 weight	Breed	0.88	0.427
	Genotype	0.21	0.814
	Genotype (breed)	0.88	0.490
CP5 weight	Breed	1.06	0.359
	Genotype	0.20	0.821
	Genotype (breed)	1.42	0.254
Carcass percentage	Breed	4.38*	0.016
	Genotype	0.04	0.964
	Genotype (breed)	1.01	0.419
CP1 percentage	Breed	15.5**	<0.001
	Genotype	3.65	0.040
	Genotype (breed)	0.94	0.455
CP2 percentage	Breed	1.94	0.164
	Genotype	6.01**	0.007
	Genotype (breed)	4.62**	0.006
CP3 percentage	Breed	2.28	0.123
	Genotype	0.14	0.871
	Genotype (breed)	1.31	0.291
CP4 percentage	Breed	2.00	0.156
	Genotype	0.85	0.440
	Genotype (breed)	0.51	0.729
CP5 percentage	Breed	0.09	0.914
	Genotype	0.11	0.896
	Genotype (breed)	0.33	0.858
Meat bone ratio	Breed	1.46	0.251
	Genotype	1.50	0.242
	Genotype (breed)	0.15	0.969

CP1 = Cut point 1, CP2 = Cut point 2, CP3 = Cut point 3, CP4 = Cut point 4, CP5 = Cut point 5, different superscripts in the same row indicate significance (p < 0.05);

* p = 0.05,

** p = 0.01. *MC4R =* Melanocortin-4 receptor, NZW = New Zealand White, RW = Reference weight; Bold font = Significant value than others.

### Breed effects on carcass characteristics

The breed effect was also significant in terms of carcass traits. As shown in Table 3, the carcass percentage differed significantly (p < 0.05) among the NZW, Hyla, and Hycole breeds. More strikingly, the percentage of CP2 varied significantly (p < 0.01) between the breeds, suggesting inherent genetic or physiological differences in muscle deposition and fat distribution. These differences highlight the importance of considering breed-specific genetic backgrounds when evaluating the effects of candidate genes on meat production.

### Genotype × breed interactions

The interaction between genotype and breed had a significant impact on CP2 weight and percentage, indicating that the *MC4R* gene effect is not uniform across breeds. Each genotype showed distinct performance in different breeds, suggesting possible gene-by-breed interactions that influence carcass quality. For instance, in one breed, a specific genotype may result in superior meat yield, whereas the same genotype may not confer the same advantage in another breed.

## DISCUSSION

### Genetic marker applications in rabbit breeding

Genetic marker research has proven to be an effective tool for determining appropriate breeding strategies and mating systems, particularly by enabling the selection of rabbits with desirable traits to serve as parents for future generations [19]. This approach can enhance the productivity and genetic progress of rabbit populations. El-Sabrout and Aggag [14] and Milisits *et al*. [20] have reported a significant association between different genotypes of the *MC4R* gene and variations in body weight and carcass characteristics. Although their study focused on chickens, similar genetic influences may be observed in rabbits.

### Carcass traits influenced by MC4R genotypes

In terms of carcass traits, CP1 and CP2 were identified as the primary commercial forequarter cuts in rabbits. According to Chodová *et al*. [21], the forepart of the rabbit exhibits the highest tissue growth rate, significantly influencing overall growth patterns and altering carcass composition ratios. Genetic factors play a critical role in determining carcass yield and quality. In addition, Sulaeman *et al*. [22] emphasized that both fore and hind limb weights are significantly affected by the breed and genotype of the rabbits, reinforcing the importance of genetic selection in breeding programs aimed at improving meat production and carcass traits in commercial rabbit farming. The type of rabbit, environment, live weight, and nutrients in the feed were all highly dependent on carcass traits and carcass percentage. Based on the studies by Pugliese and Sirtori [23] and Kamila *et al*. [24], the breed is a major determinant in the growth and quality of carcasses in rabbits, such as NZW, which are known to have good growth performance. According to El-Sabrout [25], the effect of genetic variability on the appearance and quality of meat from different species has been studied, where genetics significantly affect carcass traits.

### Performance of GG genotype in CP2 traits

The analysis revealed significant associations between MC4R genotypes and CP2 traits, with GG-genotype rabbits demonstrating superior performance in both CP2 weight (0.12 ± 0.03 kg) and percentage (10.67 ± 1.53%) compared to AG and AA genotypes (Table 2). This dose-dependent pattern suggests that the G allele may enhance carcass yield in the thoracic-lumbar region, potentially through the established role of MC4R in regulating energy metabolism and muscle deposition. Although these findings align with previous reports of the influence of MC4R on growth traits in rabbits, the significant genotype × breed interaction (Table 3) indicates that the magnitude of the effect may vary across genetic backgrounds, highlighting the need for breed-specific considerations in MAS programs aimed at improving forequarter meat yield.

### Role of MC4R in feed intake regulation

The *MC4R* gene plays a crucial role in regulating feed intake in the hypothalamus through the melanocortin signaling pathway. The *MC4R* gene is considered a strong candidate for identifying genetic factors that contribute to variability in growth performance. Mutations or polymorphisms in MC4R can alter receptor function, ultimately affecting feed intake and growth in rabbits [26]. This pathway is activated by the hormones leptin and insulin, which stimulate pro-opiomelanocortin (POMC) neurons in the arcuate nucleus [27]. These neurons then produce α-melanocyte-stimulating hormone, which activates MC4R in the paraventricular nucleus, leading to anorexigenic effects that reduce feed intake. Conversely, another set of neurons expresses neuropeptide Y andagouti-related peptide (AgRP), which act as antagonists to this system. AgRP binds to MC4R and inhibits its activity, promoting orexigenic signals that increase feed intake. In addition, the type of endoproteolytic enzymes present in POMC neurons determines the production of peptides such as α-melanocyte stimulating hormone and β-endorphins, thereby influencing the balance between appetite stimulation and suppression [28]. This complex interaction ultimately regulates rabbit feeding behavior and energy homeostasis.

### Mechanisms of MC4R action in growth and carcass development

As a key gene in the regulation of energy balance, MC4R can serve as an important genetic marker for rabbit growth performance. According to El-Sabrout and Aggag [14] and Adamska-Patruno *et al*. [29], a significant correlation exists between SNPs within the *MC4R* gene and variations in body weight among rabbits [14, 30]. This suggests that MC4R genotype-based selection could be beneficial for improving growth rates. Rabbits with a higher body weight typically exhibit greater muscle mass and fat deposition, traits that are closely associated with carcass quality. Sam *et al*. [30] reported that MC4R activity may contribute to muscle development and fat storage, thereby influencing energy metabolism and promoting the production of heavier carcasses with a higher proportion of edible meat. Furthermore, a previous study by Osaiyuwu *et al*. [31] demonstrated that live body weight was significantly and positively correlated with several carcass traits across four different crossbred groups (NZW × NZW, Chincilla [CHA] × CHA, NZW × CHA, and CHA × NZW). These findings underscore the potential of using MC4R as a selection marker in breeding programs aimed at enhancing rabbit carcass yield.

### Energy metabolism and feed efficiency

The *MC4R* gene also plays a critical role in energy metabolism regulation. Efficient use of energy is essential for optimal growth and carcass development. Feed quality and feed consumption efficiency directly influence the development of muscle and body tissues, which in turn contribute to increased carcass weight [32, 33]. Rabbits with larger body weights typically have higher nutritional requirements to sustain maintenance and growth, and their faster growth rates are often associated with greater carcass yields. Therefore, enhancing feed efficiency is a key strategy to maximize carcass production in rabbit farming. According to Wang *et al*. [34], *MC4R* gene expression occurs in the hypothalamus, where it plays a central role in regulating appetite and feeding behavior [34]. This gene modulates the balance between orexigenic (appetite-stimulating) and anorexigenic (appetite-suppressing) signals, thereby influencing body weight gain (BWG) and growth performance. When properly managed through genetic selection and nutritional strategies, the interaction between MC4R activity and feeding behavior can improve growth rates and carcass traits. These insights highlight the importance of integrating genetic markers, such as MC4R, in breeding programs aimed at enhancing feed efficiency and meat production in rabbits.

## CONCLUSION

This study demonstrated a clear association between a SNP in the *MC4R* gene at position 519 (G→A) and important carcass traits in three commercial rabbit breeds, NZW, Hyla, and Hycole, raised under tropical conditions. Three genotypes (AA, AG, and GG) were identified, with the GG genotype exhibiting superior performance in key parameters, including carcass percentage (57%), CP2 weight (0.12 ± 0.03 kg), and percentage (10.67 ± 1.53%). AG genotype rabbits recorded the highest retail weight, suggesting enhanced muscle development. Significant genotype × breed interactions for CP2 traits indicated that the genetic effect of MC4R is breed-dependent, emphasizing the importance of tailoring selection strategies to specific genetic backgrounds.

The identification of the MC4R 519G→A SNP as a potential genetic marker offers a valuable tool for MAS in rabbit breeding. Incorporating this marker into selection programs can enhance carcass yield, improve meat quality, and optimize forequarter meat production, particularly in tropical systems where environmental pressures and feed resources differ from temperate regions. This is one of the first investigations to evaluate MC4R polymorphism in multiple commercial rabbit breeds under tropical conditions, integrating detailed carcass trait analysis with genotype–breed interaction effects. The use of standardized carcass processing and consistent measurement protocols increased data reliability.

However, the study was limited by a relatively small sample size (n = 35), which may reduce statistical power for detecting more subtle genotype effects. In addition, environmental and management factors, though standardized within the study, may vary in commercial production systems, potentially influencing genotype expression. Larger-scale studies across diverse production environments are needed to validate the observed associations. Further research should integrate MC4R genotyping with other candidate genes involved in growth, metabolism, and meat quality, as well as genomic selection models to improve predictive accuracy. Longitudinal studies could also assess how MC4R effects persist across growth stages and reproductive cycles.

The findings confirm the role of MC4R as a candidate gene influencing carcass yield and composition in commercial rabbits, with the G allele contributing positively to meat deposition. Incorporating MC4R-based selection into breeding programs can accelerate genetic progress toward higher meat productivity in tropical rabbit production, contributing to food security and the economic sustainability of small-to medium-scale rabbit enterprises.

## AUTHORS’ CONTRIBUTIONS

AS and SS: Conception; DAL, FTK, and MHK: Methodology. FTK and DAL: Data Analysis. FM: Validation; AS, FTK, FM, and DAL: Writing Manuscript-originial. MHK and SS: Writing Manuscript review/revision. All authors have read and approved the final manuscript.
